# Comparison of broth microdilution and Etest^®^ methods for susceptibility testing of amphotericin B in *Candida auris*

**DOI:** 10.1093/mmy/myaf019

**Published:** 2025-03-03

**Authors:** Domenica G De Luca, Xiao Rui Li, David C Alexander, Tanis C Dingle, Philippe J Dufresne, Linda M Hoang, Julianne V Kus, Caroline Sheitoyan-Pesant, Amrita Bharat

**Affiliations:** National Microbiology Laboratory Branch, Public Health Agency of Canada, Winnipeg, MB, R3E 3R2, Canada; Department of Medical Microbiology and Infectious Diseases, University of Manitoba, Winnipeg, MB, R3E 0J9, Canada; National Microbiology Laboratory Branch, Public Health Agency of Canada, Winnipeg, MB, R3E 3R2, Canada; Department of Medical Microbiology and Infectious Diseases, University of Manitoba, Winnipeg, MB, R3E 0J9, Canada; Cadham Provincial Laboratory, Diagnostic Services, Shared Health, Winnipeg, MB, R3E 3J7, Canada; Alberta Precision Laboratories – Public Health Laboratory, Calgary, AB, T2N 4W4, Canada; Department of Pathology and Laboratory Medicine, University of Calgary, Calgary, AB, T2N 1N4, Canada; Laboratoire de santé publique du Québec, Sainte-Anne-de-Bellevue, QC, H9X 3R5, Canada; BC Centre for Diseases Control, Vancouver, BC, V5Z 4R4, Canada; Public Health Ontario, Toronto, ON, M5G 1M1, Canada; Department of Laboratory Medicine and Pathobiology, University of Toronto, Toronto, ON, M5S 3K3, Canada; Centre hospitalier universitaire Dr-Georges-L.-Dumont, Moncton, NB E1C 2Z3, Canada; National Microbiology Laboratory Branch, Public Health Agency of Canada, Winnipeg, MB, R3E 3R2, Canada; Department of Medical Microbiology and Infectious Diseases, University of Manitoba, Winnipeg, MB, R3E 0J9, Canada

**Keywords:** *Candida auris*, amphotericin B, Etest, broth microdilution, antifungal susceptibility testing

## Abstract

Amphotericin B remains an important treatment for multidrug-resistant *Candida auris*. Antifungal susceptibility testing of amphotericin B in *C. auris* can vary depending on the methodology used. Here, we compared the Etest method and the Clinical and Laboratory Standards Institute broth microdilution reference method for amphotericin B against 60 clinical *C. auris* isolates from the four major clades. The minimum inhibitory concentrations differed significantly by method (*P*-value, <.0001), and discrepancies were observed in the interpretation of resistance (categorical agreement, 88.3%; very major error, 33.3%). Broth microdilution may represent a more conservative approach for detecting amphotericin B resistance in *C. auris*.

*Candida auris* is a multidrug-resistant fungal pathogen that is associated with life-threatening invasive infections and numerous outbreaks in healthcare settings worldwide.^1^ Since the majority of *C. auris* isolates are resistant to fluconazole and, in some cases, also amphotericin B (AMB) and/or echinocandins, having antifungal susceptibility testing (AFST) methodologies that produce accurate and reliable minimum inhibitory concentrations (MICs) are critical for guiding therapeutic decisions and monitoring rates of antifungal drug resistance.^2–4^ Although echinocandins are considered first-line agents for invasive candidiasis, AMB may be utilized as an alternative therapeutic for multidrug-resistant *C. auris* infections.^5^ Based on the tentative breakpoint of resistance (≥ 2 µg/ml) established by the United States Centers for Disease Control (US CDC), ~ 30%–50% of *C. auris* isolates are resistant to AMB.^6,7^

More recently, the Clinical and Laboratory Standards Institute (CLSI) has warned that AMB susceptibility testing results should be interpreted with caution as there is significant variation in the distribution of MICs across different AFST methodologies in *C. auris*.^8^ For example, MICs determined using the Vitek-2 cards or Sensititre YeastOne panels for AMB susceptibility testing in *C. auris* were falsely elevated in comparison to the broth microdilution (BMD) reference method, resulting in overestimation of AMB resistance.^9–12^ Previous research has suggested the strip gradient diffusion Etest method may be more sensitive in detecting AMB resistance in *Candida* species.^13–15^

In this study, we evaluated the performance of the Etest method compared to the CLSI BMD reference method for AMB susceptibility testing in *C. auris*. MICs for AMB were measured by both methods against 60 clinical isolates of *C. auris* from cases of infection or colonization reported to the Public Health Agency of Canada by provincial public health laboratories between 2012 and October 2024 (Table 1). The isolates were representative of the four major clades of *C. auris* (Clade I, *n* = 34; Clade II, *n* = 8; Clade III, *n* = 13; and Clade IV, *n* = 5) (Table 1). The fifth and sixth minor clades discovered elsewhere have not been identified in Canada to date. The isolates were recovered from various body sites including axilla/groin/nares (*n* = 19), ear (*n* = 11), blood (*n* = 8), leg/toe (*n* = 6), respiratory tract (*n* = 4), urine (*n* = 4), sputum (*n* = 2), and wound (*n* = 2) and one isolate each from unspecified drainage, peritoneal fluid, urinary catheter, and unknown (Table 1).

**Table 1.** Antifungal minimum inhibitory concentrations (MICs) of amphotericin B (AMB) against *Candida auris* (*n* = 60) by broth microdilution and Etest.

Clade	Isolate	Region in Canada^1^	Year	Specimen type	Broth microdilution MIC (µg/ml)^2^	Etest MIC (µg/ml)^2^	No. of two-fold dilution difference in MICs
I	N18-01797	West	2018	Axilla/groin/nares	**2**	1	1
	N18-01798	West	2018	Axilla/groin/nares	**2**	1	1
	N18-01799	West	2018	Toe	**2**	1	1
	N18-01800	West	2018	Blood	**2**	**2**	–
	N18-01801	West	2018	Trachea	**2**	**2**	–
	N18-01802	West	2018	Drainage	**2**	**2**	–
	N18-01914	West	2018	Axilla/groin/nares	**2**	**2**	–
	N18-02487	Central West	2017	Ear	**2**	**2**	–
	N18-02621	West	2018	Axilla/groin/nares	**2**	**2**	–
	N19-03893	Central West	2019	Toe	**2**	**2**	–
	N20-01201	West	2020	Axilla/groin/nares	**2**	**2**	–
	N20-01831	Central East	2020	Blood	**2**	**2**	–
	N21-00107	Central East	2020	Axilla/groin/nares	0.5	0.25	1
	N22-02136	West	2022	Leg	0.5	0.5	–
	N22-02605	Central East	2022	Bronchial wash	0.5	0.25	1
	N22-02606	Central East	2022	Axilla/groin/nares	1	0.5	1
	N22-02862	Central East	2022	Blood	**2**	**2**	–
	N22-02863	Central East	2022	Axilla/groin/nares	**2**	**2**	–
	N22-02985	Central East	2022	Unknown	0.5	0.25	1
	N22-03030	West	2022	Blood	1	0.25	2
	N22-03211	Central East	2022	Urine	1	0.5	1
	N23-00866	West	2023	Wound	1	0.5	1
	N23-00937	Central East	2023	Urine	**2**	**2**	–
	N23-01498	Central East	2023	Sputum	1	0.5	1
	N23-02468	Central East	2023	Axilla/groin/nares	**2**	1	1
	N23-02469	Central East	2023	Urine	**2**	**2**	–
	N24-00055	West	2023	Axilla/groin/nares	**2**	1	1
	N24-00587	Central East	2024	Axilla/groin/nares	**2**	1	1
	N24-01105	West	2024	Axilla/groin/nares	0.25	0.25	–
	N24-01530	West	2024	Leg	**2**	1	1
	N24-03217	West	2024	Urine	1	0.25	2
	N24-03372	Central East	2024	Wound	1	0.25	2
	N24-03374	Central East	2024	Ear	0.5	0.12	2
	N24-03460	West	2024	Axilla/groin/nares	1	0.5	1
II	N18-02485	West	2014	Ear	0.5	0.5	–
	N19-02739	West	2019	Ear	0.5	0.5	–
	N19-03592	West	2019	Ear	0.5	0.5	–
	N20-01951	West	2019	Ear	0.5	0.5	–
	N21-00557	East	2021	Ear	0.25	0.25	–
	N21-02638	West	2021	Ear	0.5	0.25	1
	N22-00029	West	2021	Ear	1	0.5	1
	N24-03373	Central East	2024	Ear	0.5	0.25	1
III	N18-02486	Central East	2012	Ear	1	0.5	1
	N19-00171	West	2019	Sputum	0.5	0.5	–
	N19-00216	West	2019	Axilla/groin/nares	0.5	0.5	–
	N19-01047	West	2018	Bronchial wash	1	0.5	1
	N20-01343	Central East	2020	Leg	1	0.5	1
	N22-01213	Central East	2022	Bronchoalveolar lavage	**2**	**2**	–
	N22-01518	Central East	2022	Axilla/groin/nares	1	0.5	1
	N22-01519	Central East	2022	Urinary catheter	0.25	0.5	1
	N22-03212	Central East	2022	Blood	1	0.25	2
	N23-01496	Central East	2023	Axilla/groin/nares	0.5	0.5	–
	N23-01497	Central East	2023	Axilla/groin/nares	1	0.5	1
	N23-03371	East	2023	Axilla/groin/nares	0.5	0.25	1
	N24-00858	Central East	2024	Axilla/groin/nares	1	0.25	2
IV	N18-02509	Central East	2014	Blood	1	0.5	1
	N18-02510	Central East	2017	Leg	0.5	0.25	1
	N18-02511	Central East	2014	Blood	0.5	0.25	1
	N18-02513	Central East	2014	Blood	0.5	0.12	2
	N19-00135	Central East	2015	Peritoneal fluid	0.5	0.25	1

1 Regions in Canada were defined as West (British Columbia or Alberta), Central West (Saskatchewan or Manitoba), Central East (Ontario or Quebec), and East (New Brunswick, Newfoundland, Nova Scotia, or Prince Edward Island).

2 Boldface indicates AMB resistance according to the tentative breakpoint of ≥ 2 µg/ml established by the United States Centers for Disease Control.

AFST by BMD was carried out using a custom antifungal panel (Thermo Oxoid, Oakwood Village, OH) with RPMI media following the CLSI M27-Ed4 guidelines, with a slight temperature modification.^16^ BMD panels were incubated at 37°C rather than 35°C because this provided more reproducible results for the control strains. The Etest was performed on RPMI agar according to the manufacturer's instructions (bioMérieux Canada, St-Laurent, QC). BMD panels and Etest strips were incubated at 37°C and evaluated for growth following 24-hour and 48-hour incubations, respectively. AMB MIC concentrations ranged from 0.03 to 16 μg/ml for BMD and 0.002 to 32 μg/ml for the Etest. C*andida krusei* ATCC 6258 and *Candida parapsilosis* ATCC 22019 were used as quality control strains. Testing was repeated for isolates that yielded discordant interpretations between methods. The MICs for AMB were determined as the lowest drug concentration that completely inhibited growth. This was the concentration that produced an optically clear well for BMD or where the ellipse of the inhibition zone intersected the Etest strip (Supplementary Fig. 1).^16,17^ Isolates with an AMB MIC value of ≥ 2 µg/ml were classified as resistant. As recommended, Etest results that fell between standard two-fold dilutions were rounded up to the next higher dilution. For example, an Etest result of 1.5 µg/ml was rounded to 2.0 µg/ml.

Of the 60 *C. auris* isolates tested, AMB MICs were identical by both methods for 24 isolates (40.0%), one dilution higher by BMD for 28 isolates (46.7%), one dilution higher by Etest for a single isolate (1.7%), and two dilutions higher by BMD for seven isolates (11.7%) (Table 1). More isolates were resistant to AMB by BMD (*n* = 21) compared to the Etest (*n* = 14) (Table 1). A narrow range of MICs was obtained by both methods (BMD, 0.25–2 µg/ml; Etest, 0.12–2 µg/ml) and MICs differed significantly by method (paired *t* test; *P*-value, <.0001).

Essential agreement (EA) and categorical agreement (CA) were assessed using BMD as the gold standard. MICs were within two two-fold dilutions for all 60 clinical isolates tested (EA, 100%). Both methods produced the same category of susceptibility for 53/60 isolates (CA, 88.3%). Of the 21 isolates that were resistant by BMD, seven isolates were false-susceptible by Etest, resulting in a very major error rate of 33.3%. No instances of major error (ME, false resistance) were identified. A previous study of 31 clinical isolates showed comparable EA (100%) and CA (90.3%) between BMD and Etest methods with three MEs (9.7%).^11^ Lower EA was found in a larger study of 90 clinical *C. auris* isolates (EA, 81.0%) with 14 isolates (15.6%) resistant by BMD and one isolate (1.1%) by Etest.^9^ In a larger study of 400 clinical isolates of *C. auris*, 107 (26.8%) were resistant by BMD while only 22 (5.5%) were resistant by Etest.^18^ Although some of these previous studies do not explicitly report the agreement and/or rates of error, there are clear discrepancies in the categorization of AMB resistance between the Etest and BMD methods.

AMB-resistant *C. auris* isolates are primarily associated with Clades I and IV with resistance rarely observed in Clades II and III.^19^ Our results were consistent with this observation as the majority of AMB-resistant isolates were in Clade I (BMD, *n* = 20; Etest, *n* = 13). Clade III had a single isolate determined to be resistant by both methods. Resistant isolates were not detected by either method in Clades II or IV.

Since mechanisms of AMB resistance are not well characterized, we cannot determine with certainty if the seven *C. auris* isolates that were resistant by BMD but susceptible by Etest truly represent cases of AMB resistance. A single frameshift variant (YY98V*) in *ERG6* is the only confirmed mechanism of AMB resistance in *C. auris*.^20^ In our collection, all isolates were wild-type for *ERG6* and therefore had unknown mechanisms of potential AMB resistance. Other potential mechanisms that may contribute to AMB resistance involve lipid alterations, chromatin modifications, and DNA damage homeostasis.^21,22^ A better understanding of molecular mechanisms of AMB resistance in *C. auris* is necessary to guide phenotypic susceptibility testing.

All AMB-resistant *C. auris* isolates in this study had an MIC of 2 µg/ml, which is also the resistance breakpoint, and all isolates had a narrow range of MICs. The tight distribution of AMB MICs with both BMD and Etest methods (Fig. 1) highlights the difficulty of AMB breakpoint determination and resistance categorization. When the variability of the tests is taken into account, there is overlap between susceptible and resistant isolate populations. The tentative AMB breakpoint proposed by the US CDC is based on data from a pharmacokinetic/pharmacodynamic study in a murine model of infection.^7^

**Figure 1.**
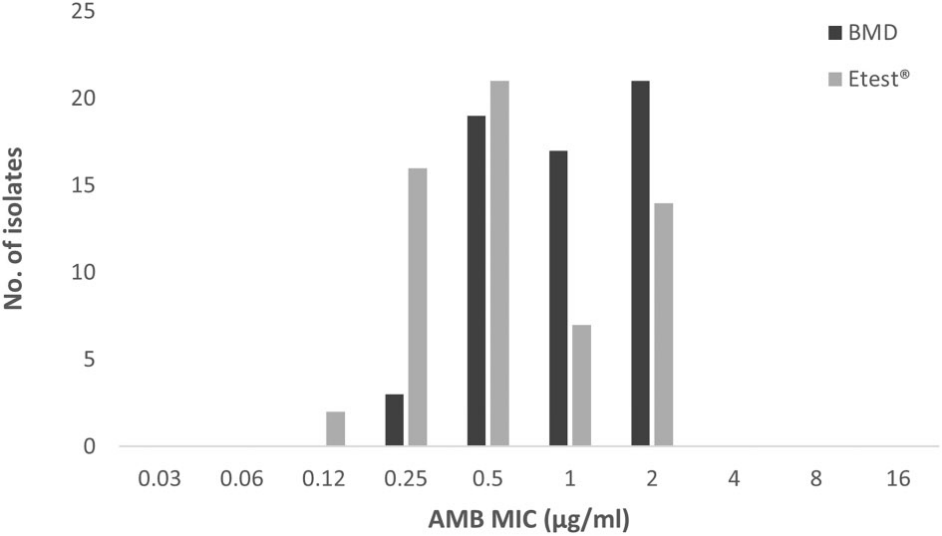
Distribution of amphotericin B (AMB) minimum inhibitory concentrations (MICs) for *Candida auris* (*n* = 60) by broth microdilution (BMD, dark grey) and Etest (light grey). Both BMD and Etest methods demonstrated a unimodal distribution of AMB MICs.

Accurate AFST is critical for guiding therapeutic treatment for patients with *C. auris* infection. The results from our study suggest that BMD is optimal for AMB susceptibility testing in *C. auris*. While BMD and Etest methods were comparable, the Etest detected fewer cases of potential AMB resistance, possibly underestimating resistance. In this study, BMD represented a more conservative approach for detecting AMB resistance in *C. auris*.

## Supplementary Material

myaf019_Supplemental_File

## References

[1] WHO fungal priority pathogens list to guide research, development and public health action, World Health Organization. Published October 5, 2022. https://www.who.int/publications/i/item/9789240060241. (Date accessed: March 10, 2024).

[2] Chowdhary A, Prakash A, Sharma C, et al. A multicentre study of antifungal susceptibility patterns among 350 *Candida auris* isolates (2009–17) in India: Role of the *ERG11* and *FKS1* genes in azole and echinocandin resistance. J Antimicrob Chemother. 2018; 73(4): 891–899.. 10.1093/jac/dkx480 29325167

[3] Lyman M, Forsberg K, Sexton DJ, et al. Worsening spread of *Candida auris* in the United States, 2019 to 2021. Ann Intern Med. 2023; 176(4): 489–495.. 10.7326/M22-3469 36940442 PMC11307313

[4] Berkow EL, Lockhart SR, Ostrosky-Zeichner L. Antifungal susceptibility testing: Current approaches. Clin Microbiol Rev. 2020; 33(3): e00069–19.. 10.1128/CMR.00069-19 32349998 PMC7194854

[5] Pappas PG, Kauffman CA, Andes DR, et al. Clinical practice guideline for the management of candidiasis: 2016 update by the Infectious Diseases Society of America. Clin Infect Dis. 2016; 62(4): e1–e50.. 10.1093/cid/civ933 26679628 PMC4725385

[6] Lockhart SR, Etienne KA, Vallabhaneni S, et al. Simultaneous emergence of multidrug-resistant *Candida auris* on 3 continents confirmed by whole-genome sequencing and epidemiological analyses. Clin Infect Dis. 2017; 64(2): 134–140.. 10.1093/cid/ciw691 27988485 PMC5215215

[7] United States Centers for Disease Control . Antifungal susceptibility testing for C*andida auris*. Published April 23, 2024. https://www.cdc.gov/candida-auris/hcp/laboratories/antifungal-susceptibility-testing.html. (Date Accessed: March 13, 2024).

[8] Clincal and Laboratory Standards Institute . AST News Update June 2022: Hot Topic *Candida auris* update: Method variability with amphotericin B susceptibility testing. Published June 11, 2022. https://clsi.org/about/blog/ast-news-update-june-2022-hot-topic. (Date accessed: March 20, 2024).

[9] Kathuria S, Singh PK, Sharma C, et al. Multidrug-resistant *Candida auris* misidentified as *Candida haemulonii*: Characterization by matrix-assisted laser desorption ionization-time of flight mass spectrometry and DNA sequencing and its antifungal susceptibility profile variability by Vitek 2, CLSI broth microdilution, and Etest method. J Clin Microbiol. 2015; 53(6): 1823–1830.. 10.1128/JCM.00367-15 25809970 PMC4432077

[10] Siopi M, Peroukidou I, Beredaki M-I, et al. Overestimation of amphotericin B resistance in *Candida auris* with Sensititre YeastOne antifungal susceptibility testing: A need for adjustment for correct interpretation. Microbiol Spectr. 2023; 11(3): e0443122. 10.1128/spectrum.04431-22 37036351 PMC10269614

[11] Ceballos-Garzon A, Garcia-Effron G, Cordoba S, et al. Head-to-head comparison of CLSI, EUCAST, Etest and VITEK®2 results for *Candida auris* susceptibility testing. Int J Antimicrob Agents. 2022; 59(4): 106558. 10.1016/j.ijantimicag.2022.106558 35227828

[12] Siopi M, Pachoulis I, Leventaki S, et al. Evaluation of the Vitek 2 system for antifungal susceptibility testing of *Candida auris* using a representative international panel of clinical isolates: Overestimation of amphotericin B resistance and underestimation of fluconazole resistance. J Clin Microbiol. 2024; 62(4): e0152823. 10.1128/jcm.01528-23 38501836 PMC11005389

[13] Pfaller MA, Messer SA, Bolmström A. Evaluation of Etest for determining *in vitro* susceptibility of yeast isolates to amphotericin B. Diagn Microbiol Infect Dis. 1998; 32(3): 223–227.. 10.1016/s0732-8893(98)00120-5 9884840

[14] Peyron F, Favel A, Michel-Nguyen A, Gilly M, Regli P, Bolmström A. Improved detection of amphotericin B-resistant isolates of *Candida lusitaniae* by Etest. J Clin Microbiol. 2001; 39(1): 339–342.. 10.1128/JCM.39.1.339-342.2001 11136795 PMC87726

[15] Wanger A, Mills K, Nelson PW, Rex JH. Comparison of Etest and National Committee for Clinical Laboratory Standards broth macrodilution method for antifungal susceptibility testing: Enhanced ability to detect amphotericin B-resistant *Candida* isolates. Antimicrob Agents and Chemother. 1995; 39(11): 2520–2522.. 10.1128/aac.39.11.2520 8585737 PMC162976

[16] Clinical and Laboratory Standards Institute . Reference Method for Broth Microdilution Antifungal Susceptibility Testing of Yeasts. 4th edition. CLSI standard M27. Wayne PA, USA,: Clinical and Laboratory Standards Institute, 2017.

[17] Wiederhold NP . Antifungal suseptibility testing: A primer for clinicians. Open Forum Infect Dis. 2021;8(11): ofab444. 10.1093/ofid/ofab444 34778489 PMC8579947

[18] Maphanga TG, Naicker SD, Kwenda S, et al. *In vitro* antifungal resistance of *Candida auris* isolates from bloodstream infections, South Africa. Antimicrob Agents and Chemother. 2021; 65(9): e0051721. 10.1128/AAC.00517-21 34228535 PMC8370198

[19] Chow NA, Muñoz JF, Gade L, et al. Tracing the evolutionary history and global expansion of *Candida auris* using population genomic analyses. mBio. 2020; 11(2): e03364–19.. 10.1128/mBio.03364-19 32345637 PMC7188998

[20] Rybak JM, Barker KS, Muñoz JF, et al. *In vivo* emergence of high-level resistance during treatment reveals the first identified mechanism of amphotericin B resistance in *Candida auris*. Clin Microbiol Infect. 2022; 28(6): 838–843.. 10.1016/j.cmi.2021.11.024 34915074 PMC9467277

[21] Shivarathri R, Jenull S, Chauhan M, et al. Comparative transcriptomics reveal possible mechanisms of amphotericin B resistance in *Candida auris*. Antimicrob Agents Chemother. 2022; 66(6): e0227621. 10.1128/aac.02276-21 35652307 PMC9211394

[22] Carolus H, Pierson S, Muñoz JF, et al. Genome-wide analysis of experimentally evolved *Candida auris* reveals multiple novel mechanisms of multidrug resistance. mBio. 2021; 12(2): e03333–20.. 10.1128/mBio.03333-20 33820824 PMC8092288

