# Supplementary material for: Comparison of broth microdilution and Etest® methods for susceptibility testing of amphotericin B in Candida auris
Source: Med Mycol. 2025 Mar 3;63(3):myaf019. doi: 10.1093/mmy/myaf019 (PMC11899573; doi:10.1093/mmy/myaf019)
Supplement: myaf019_Supplemental_File [file myaf019_supplemental_file.pdf]

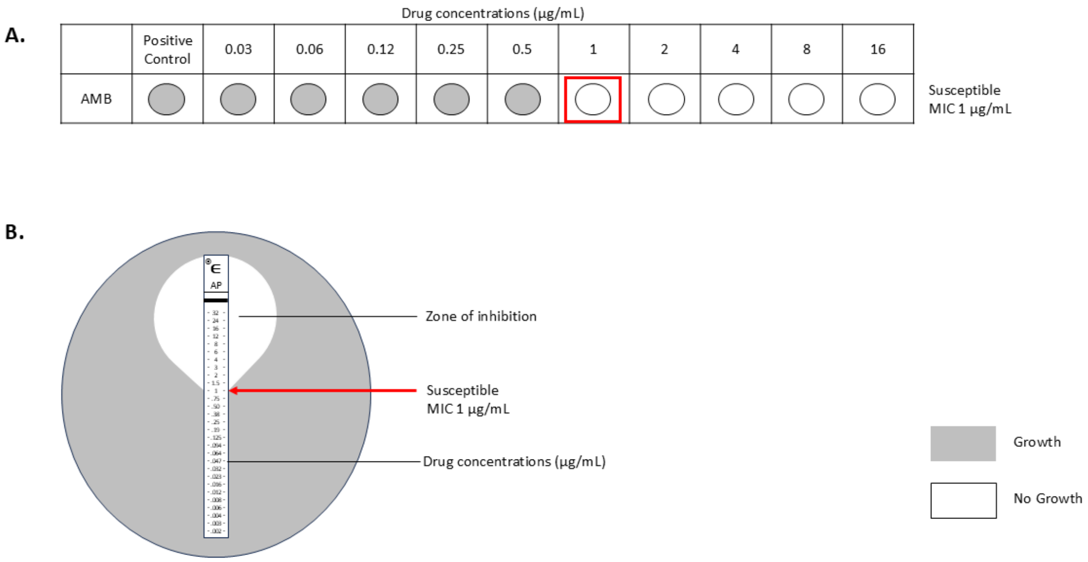

Supplementary Figure 1. Amphotericin B susceptibility testing by broth microdilution (BMD) and Etest methods in *Candida auris*. (A) For BMD, the minimum inhibitory concentration (MIC) is the lowest drug concentration that produces an optically clear well. (B) For Etest, the MIC is where the ellipse of the zone of inhibition intersects the Etest strip. The red box (A) and arrow (B) indicates the MIC.
